# Indirect Application of Intense Pulsed Light Induces Therapeutic Effects on Experimental Murine Meibomian Gland Dysfunction

**DOI:** 10.3389/fmed.2022.923280

**Published:** 2022-06-02

**Authors:** Luoying Xie, Wenjing Song, Wenhui Dong, Yingsi Li, Shudi Chen, Xiaona Sun, Meiting Huang, Yu Cheng, Yuan Gao, Songlin Yang, Xiaoming Yan

**Affiliations:** Department of Ophthalmology, Peking University First Hospital, Beijing, China

**Keywords:** intense pulsed light, meibomian gland dysfunction, indirect effect, inflammation, photobiomodulation

## Abstract

**Purpose:**

To investigate the indirect effects of intense pulsed light (IPL) on morphological and pathological changes of the meibomian glands (MGs) in apolipoprotein E knockout (*ApoE^–/–^*) mice and explore the underlying mechanisms.

**Methods:**

*ApoE^–/–^* mice were treated with or without IPL three times below the lower eyelids and MGs were not directly exposed to irradiation. The eyelids and ocular surface were observed under a stereoscope. The morphology of MGs was examined by photographing and hematoxylin and eosin staining. Lipid droplets in MGs were examined by Oil Red O staining. The ultrastructure of meibocytes and mitochondria was observed under transmission electron microscopy. The relative gene and protein expression in MGs of upper eyelids was determined by immunostaining, Western blot, and qRT–PCR.

**Results:**

Three IPL treatments decreased the toothpaste-like plugging of orifices and thickening and irregularity of the upper and lower eyelid margins in *ApoE^–/–^* mice. The morphology of some MGs improved after IPL treatments, accompanied by increased proliferation of acinar basal cells and decreased ductal keratinization. Furthermore, the accumulation of hyperchromatic lipid droplets in the acini increased, and the lipid droplets distributed in the cells around the acini were round and small. Compared with untreated *ApoE^–/–^* mice, oxidative stress and apoptosis were downregulated by IPL treatment, accompanied by the improvements in mitochondrial structure. Further research showed that IPL treatments reduced the levels of tumor necrosis factor-alpha (TNF-α), interleukin (IL)-17A, IL-6 in MGs and inactivated nuclear factor kappa B (NF-κ B).

**Conclusion:**

Collectively, the results demonstrate that indirect effects of IPL can improve the structure and function of MGs and mitigate the progression of MGD, which may be related to the indirect effects of photobiomodulation.

## Introduction

Meibomian glands (MGs) are modified sebaceous glands within the eyelids, which synthesize and secrete meibum into the tear film, forming the outer lipid layer to delay the evaporation of aqueous tear (1). Meibomian gland dysfunction (MGD) is a chronic, diffuse malfunction of MGs, and the underlying pathophysiology of MGD has been reported to be obstruction of the MG orifices and ductal hyperkeratinization, which causes impaired secretion of meibum (1). Stasis of MG secretion in the duct leads to intraglandular cystic dilatation and acinar atrophic degeneration, ultimately resulting in tear film instability and disruption of ocular surface homeostasis (2–5).

Intense pulsed light (IPL) is an effective treatment for MGD and has been used in ophthalmology. IPL is a broad-spectrum incoherent light with wavelengths of 500–1,200 nm, which is produced by high-output xenon flash lamps (6, 7). The photons emitted by IPL can be absorbed by chromophores such as hemoglobin, thereby triggering effects such as selective photothermolysis to achieve therapeutic purposes. Clinical trials have reported that IPL can ameliorate the signs and symptoms of patients with MGD, attenuate ocular surface inflammation, improve tear film stability, eliminate *Demodex* by coagulative necrosis, and provide long-term effectiveness (8–12). Although there have been many clinical studies, the exact mechanism underlying its beneficial effects in treating MGD remains unclear. In addition to the heating effect, IPL as a type of light therapy may also have the effect of photobiomodulation (PBM). PBM utilizes light sources including low level laser (LLL), light emitting diodes (LEDs) and broadband light to emit photons in the red or near-infrared (NIR) spectrum, which are captured by endogenous chromophores to induce non-thermal effects such as photophysical or photochemical effects (13). The reported beneficial outcomes of PBM include promoting tissue regeneration and wound healing (14, 15), decreasing inflammation and pain (16, 17), and enhancing antioxidant defenses (18). We have discovered that IPL significantly reduced the levels of inflammatory factors—interleukin (IL)-17A, IL-6, and PGE2—in tears of MGD patients (10). Given that the energy of IPL for the treatment of MGD is relatively low and its spectrum includes red and NIR light (600–1,100 nm), we hypothesized that the photobiomodulatory effect of IPL also plays a role in treating MGD, especially its anti-inflammatory effect.

In clinical treatment, IPL is usually applied to the skin below the lower eyelids and the eyes are protected by eye shields. It is worth noting that although MGs are not directly exposed to light, MG scores and MG secretion functions were improved in both the upper and lower eyelids (19, 20). This may indicate that the therapeutic effect of IPL can be obtained through target tissues, even if the MGs are not directly exposed to the irradiation range of IPL; that is, IPL treatment is highly likely to have the indirect effect of irradiation, in addition to the direct effect of irradiation. In fact, the indirect effect of light therapy has been widely observed in PBM (21–23). Hence, we proposed that the improvements in MGD due to the indirect effect of IPL may be related to PBM. Herein, we investigated the indirect effects of IPL on morphological and pathological changes in MGs and explored the possible therapeutic mechanisms of IPL by examining PBM-related inflammatory pathways.

## Materials and Methods

### Animals

To examine whether IPL treatment can attenuate the severity of MGD, we chose *ApoE^–/–^* mice as the model to observe the clinical and pathological changes after IPL treatment. Previous studies have reported that apolipoprotein E knockout (*ApoE^–/–^*) mice begin to show morphological and functional abnormalities of MGs at 5 months of age, which is attributed to hyperlipidemia (24). Therefore, we treated 6-month-old *ApoE^–/–^* mice with three sessions of IPL to observe whether MGD improved. The experiments were approved by the Laboratory Animal Ethics Committee of Peking University First Hospital and were performed in accordance with the ARVO Statement for the Use of Animals in Ophthalmic and Vision Research.

Male C57BL/6 mice and *ApoE^–/–^* mice were purchased from Huafukang Biotechnology Co., Ltd. (Beijing, China) and raised in the animal center of Peking University First Hospital. All mice were fed a standard-fat diet and were divided into three subgroups: the wild-type (WT) group (C57BL/6 mice), model group (*ApoE^–/–^* mice) and IPL-treated group (*ApoE^–/–^* mice received three IPL treatments). Clinical parameters of all the mice were examined 2 weeks after each IPL treatment.

### Intense Pulsed Light Treatment

After the mice were anesthetized, the hair on both sides of their face was shaved. Prior to treatment, a cooling ultrasound gel was administered to the irradiation area and eyes were shielded from the light stimulus.

An Eyesis IPL system (MDC (TianJin) Co., Ltd. China) was used for treatment. The intensity of the IPL treatment was 12 J/cm^2^ and 1 pulse was applied to both sides with three treatment sessions at 2-week intervals. IPL therapy was administered to the skin 3 mm far from the lid margin of the lower eyelid and the irradiation area was 1 square centimeter of the mouse face. The irradiance and frequency of radiation were based on clinical application and the data from our preliminary experiments.

### Meibomian Gland and Ocular Surface Examination

Clinical parameters, including plugged orifice numbers, lid margin irregularity, lid margin thickening, and corneal fluorescein staining scores, were measured under a stereoscope (EZ4; Leica Microsystems, Wetzlar, Germany) at 2 weeks after each IPL treatment. All parameters were blindly assessed by a single experienced ophthalmologist (WD).

To determine plugged orifice numbers, eight orifices in the center of the upper and lower eyelid were selected respectively, and plugged orifices were counted based on previously reported criteria (25). A swollen orifice protruding from the lid margin with a toothpaste-like plug was considered to be a plugged orifice. Lid margin irregularity and thickening were scored from 0 to 2 according to previously reported criteria (26). Both upper and lower eyelid margins were evaluated. Corneal fluorescein staining was performed by instilling 1% liquid sodium fluorescein, and the eye was observed using a stereoscope under cobalt blue illumination. The staining score was evaluated according to previously reported criteria (27).

### Histology

Eyelids were harvested after the mice were sacrificed. Then, eyelid tissues were embedded in optimal cutting temperature compound, immediately frozen in liquid nitrogen, sectioned at 6 μm and kept at −80°C. Frozen sections were used for hematoxylin and eosin (H&E), oil red O (ORO), and immunofluorescence staining. Additional eyelids were immersed in formalin for fixation and then processed into paraffin blocks until sectioned (4 μm sagittal section) for immunohistochemical staining.

### Oil Red O Staining

Frozen sections were fixed with 4% paraformaldehyde for 10 min and rinsed with phosphate buffered saline (PBS). Sections were then immersed in 60% isopropanol for 30 s and stained with freshly prepared ORO solution for 15 min, followed by 60% isopropanol for 5 s. Thereafter, the sections were stained with hematoxylin and mounted with glycerol gelatin.

### Immunofluorescence Analysis

Briefly, frozen sections were dried at room temperature for 15 min and washed with PBS. Then, the sections were incubated with anti-Ki67 antibody (1:100; ab16667; Abcam, Cambridge, United Kingdom) and anti-Keratin 10 (K10) (1:100; ab76318; Abcam) for 30 min at 37°C. After washing with PBS, the sections were covered with Alexa Fluor 488-conjugated donkey anti-rabbit IgG (1:200; A21206; Invitrogen, Eugene, OR, United States) for 30 min at 37°C. Then, nuclear identification was performed with DAPI (ZLI-9557; ZSGB-Bio, Beijing, China). Fluorescent images were taken under a fluorescence microscope (Eclipse 80i; Nikon, Tokyo, Japan). The number of Ki67-positive cells and the fluorescence intensity of K10 were quantified with ImageJ 1.52a software.

### Immunohistochemistry

Paraffin sections were routinely deparaffinized and hydrated. Following antigen retrieval, the sections were washed and incubated with endogenous peroxidase blocker for 10 min. After washing with PBS and incubating with 2% bovine serum albumin (BSA) for 1 h at room temperature, the sections were subsequently incubated with anti-NADPH oxidase 4 (NOX-4) antibody (1:200; ab133303; Abcam) at 4°C overnight. The next day, the sections were washed and covered with enzyme-conjugated goat anti-rabbit IgG (PV6001; ZSGB-BIO, Beijing, China) for 1 h at room temperature, followed by visualization with a diaminobenzidine (DAB) kit (ZLI-9017; ZSGB-BIO). Afterward, the sections were counterstained with hematoxylin, dehydrated with ethanol and mounted in neutral gum. The staining was evaluated under a microscope.

### Transmission Electron Microscopy

After the mice were euthanized, MG tissues were immediately placed in 3% glutaraldehyde at 4°C overnight. After washing with PBS, they were fixed in 1% osmium tetroxide for 2 h. The specimens were then dehydrated in graded acetone, embedded in Epon, sectioned into 70–90 nm thick ultrathin sections and stained with 5% uranyl acetate and lead citrate. The sections were photographed with a transmission electron microscope (JEM-1400Flash; JEOL, Tokyo, Japan).

### Isolation of Mouse Meibomian Glands

The eyelids were harvested and imaged after the mice were euthanized. Then, the palpebral conjunctiva was scraped off the eyelids, and the tarsal plates (TPs) were isolated from the overlying muscle and surrounding tissues. The TPs were then immediately frozen with liquid nitrogen.

### Western Blot

The TPs were meticulously isolated and then placed into lysis buffer containing cocktails of protease and phosphatase inhibitor. There were 4 samples in each group, and each sample consisted of TPs from both upper eyelids of the same mouse. The same amount of protein was loaded on 12% SDS-PAGE gels for electrophoretic separation, followed by being electronically transferred to a PVDF membrane. After blocking with 5% skimmed milk, the membrane was incubated with rabbit anti-caspase-3 antibody (1:1,000; 9662; Cell Signaling Technology, Danvers, MA, United States), rabbit anti- nuclear factor kappa B (NF-κB) antibody (1:1,000; 8242; Cell Signaling Technology), rabbit anti-phospho (p)-NF-κB antibody (1:1,000; 3033; Cell Signaling Technology), and mouse anti-β-actin antibody (1:1,000; AA128; Beyotime, Shanghai, China) at 4°C overnight. Then, the blots were incubated with HRP-labeled goat anti-mouse IgG (1:1,000; A0216; Beyotime) or anti-rabbit IgG (1:1,500; 7074; Cell Signaling Technology) for 1 h at room temperature. BeyoECL Star (P0018AS; Beyotime) was used to visualize the specific bands which were imaged using a chemiluminescence imaging system (GeneGnome XRQ; Syngene, Cambridge, United Kingdom). Staining intensities were quantified with ImageJ 1.52a software.

### Quantitative Real-Time PCR

The TPs were isolated from both upper eyelids and combined as one sample, and five samples in WT and IPL-treated group and 6 samples in model group were used for Quantitative Real-Time PCR (qRT-PCR). TRIzol reagent (15596026, Invitrogen) was used for extracting RNA and its purity and concentration were measured by spectrophotometry. RNA (2 μg) was reverse transcribed into cDNA using a reverse transcription kit (4368814, Invitrogen). cDNA (10.0 ng) was added to 20 μl total reaction volume containing reverse transcription master mix (4368814; Invitrogen) and specific primers. The sequences of the primers are shown in Table 1. qRT–PCR was performed with an ABI PRISM 7500 Fluorescent Quantitative PCR system (Applied Biosystems, Foster City, CA, United States). The Ct values were recorded, and the relative expression levels of different genes were calculated using the 2^–ΔΔCT^ method with β-actin as the reference gene.

**TABLE 1:** Primer Sequences Used for qRT-PCR.

Gene	Primer	The primers sequence
Ki67	Forward	5′–ATCATTGACCGCTCCTTTAGGT–3′
	Reverse	5′–GCTCGCCTTGATGGTTCCT–3′
K10	Forward	5′–GCCTCCTACATGGACAAAGTC–3′
	Reverse	5′–GCTTCTCGTACCACTCCTTGA–3′
Nox-4	Forward	5′–TGCCTGCTCATTTGGCTGT–3′
	Reverse	5′–CCGGCACATAGGTAAAAGGATG–3′
TNF-α	Forward	5′–CAGGCGGTGCCTATGTCTC–3′
	Reverse	5′–CGATCACCCCGAAGTTCAGTAG–3′
IL-1β	Forward	5′–GAAATGCCACCTTTTGACAGTG–3′
	Reverse	5′–TGGATGCTCTCATCAGGACAG–3′
IL-6	Forward	5′–CTGCAAGAGACTTCCATCCAG–3′
	Reverse	5′–AGTGGTATAGACAGGTCTGTTGG–3′
IL-17A	Forward	5′–TCAGCGTGTCCAAACACTGAG–3′
	Reverse	5′–CGCCAAGGGAGTTAAAGACTT–3′
β-actin	Forward	5′–GGCTGTATTCCCCTCCATCG–3′
	Reverse	5′–CCAGTTGGTAACAATGCCATGT–3′

### Statistical Analysis

Statistical analysis was performed using SPSS 26.0 statistical software (IBM Corp., Armonk, NY, United States). All data are presented as means ± SD. One-way ANOVA with LSD *post-hoc* test was performed for data analysis. GraphPad Prism version 7.0 (GraphPad Software, San Diego, United States) was used for statistical analysis and graphing. Any *p*-value < 0.05 was considered statistically significant.

## Results

### Changes of Eyelid Margin and Ocular Surface After Intense Pulsed Light Treatment

**FIGURE 1:**
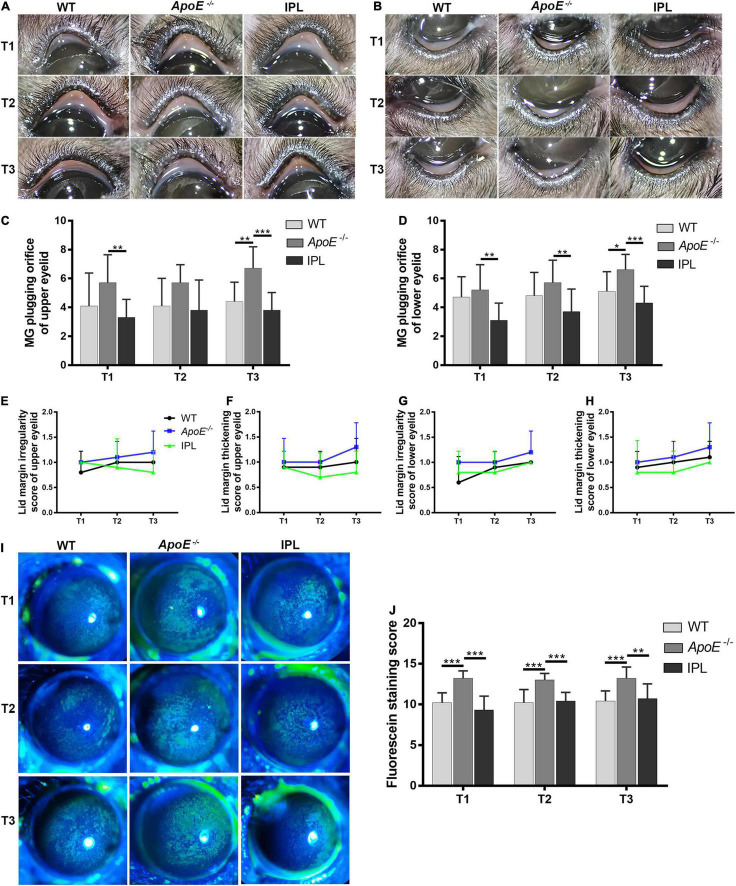
Eyelid margin and ocular surface changes after IPL treatment. Representative macrophotographs of eyelid margins from the wild type group (WT), model group (*APOE*^–/–^), and IPL-treated group (IPL) showing MG orifices at the lid margins of the upper eyelids **(A)** and lower eyelids **(B)** at two weeks after the first (T1), second (T2) and third (T3) IPL treatments. Quantitative morphometric analysis of plugged MG orifices at the lid margin of the upper eyelids **(C)** and lower eyelids **(D)** (*n* = 10/group). **(E,F)** Clinical score of lid margin irregularity and **(G,H)** lid margin thickening (*n* = 10/group). **(I)** Representative fluorescein staining images and **(J)** staining scores (*n* = 10/group). *p < 0.05, **p < 0.01, ***p < 0.001.

We evaluated the clinical manifestations of MGD in mice 2 weeks after each IPL treatment (T1, T2, T3). As shown in Figures 1A,B, eyelid margins in *ApoE^–/–^* mice showed toothpaste-like plugs at MG orifices that protruded from the margin, which were different from the orifices observed in age-matched WT mice, while the degree of orifice obstruction in both the upper and lower eyelids improved from the first IPL treatment. We counted the number of plugged orifices and found that the difference in the number of plugged orifices in the upper eyelids among the three groups at T1 and T3 time points was statistically significant (*p* < 0.05); *post-hoc* analysis found that at T1 time point, the number of plugged orifices in the IPL-treated group was lower than that in *ApoE*^–/–^ mice (*p* < 0.05), and *ApoE^–/–^* mice had more plugged orifices than WT mice and IPL-treated mice at T3 time point (*p* < 0.01, *p* < 0.001) (Figure 1C and Supplementary Table 1). In addition, at T3 time point, the number of plugged orifices in the lower eyelids of *ApoE^–/–^* mice was more than that of WT mice (*p* < 0.05), while the number of plugged orifices of IPL-treated mice was reduced at each time point compared with *ApoE*^–/–^ mice (*p* < 0.05) (Figure 1D and Supplementary Table 2).

Mice also showed other features of MGD, such as lid margin irregularity and thickening. At T2 and T3 time points, the lid margin irregularity and thickening scores of the upper and lower eyelids of *ApoE^–/–^* mice tended to increase compared with WT mice, while the scores in *ApoE^–/–^* mice treated with IPL irradiation showed a trend of decrease, although the difference was not statistically significant (Figures 1E–H).

Corneal fluorescein staining in WT mice showed age-related changes, whereas the corneal fluorescein staining score of *ApoE^–/–^* mice was higher than that of WT mice at all time points. The differences in corneal fluorescein staining scores among the three groups were statistically significant at T1, T2, and T3 time points (*p* < 0.001). At each time point, the corneal fluorescein staining scores of *ApoE^–/–^* mice was higher than that of WT mice (*p* < 0.05), and the scores of IPL-treated group was lower than that of *ApoE^–/–^* mice (*p* < 0.05) (Figures 1I,J and Supplementary Table 3).

In short, these results suggested that three IPL treatments alleviated the degree of clinical MGD-like changes and attenuated corneal epithelial damage in *ApoE^–/–^* mice.

### Intense Pulsed Light Treatment Ameliorates Meibomian Orifice Obstruction and Meibomian Gland Morphology

**FIGURE 2:**
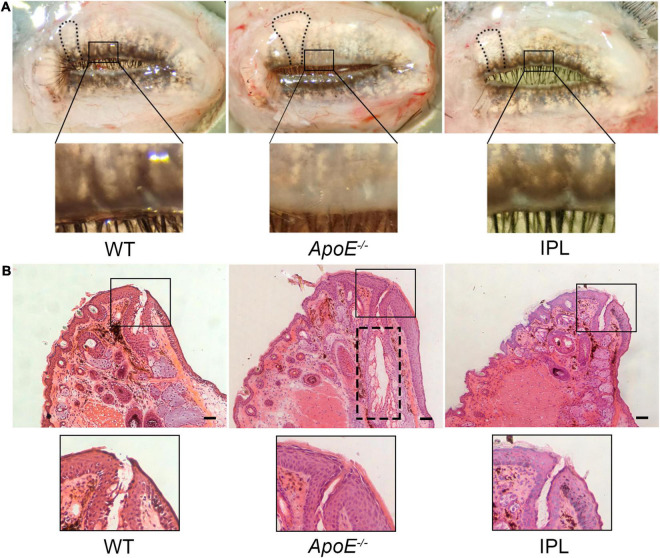
Morphology and histology of MGs after IPL treatments. **(A)** Macrophotographs of MGs in eyelids. The black frame indicates unplugged orifices in WT mice and IPL-treated mice and plugged orifices in *APOE*^–/–^ mice. The black line indicates that the morphological hypertrophy of MGs in IPL-treated mice was reduced. **(B)** H&E staining of the upper eyelids. The black frame indicates unplugged orifice in WT mice and IPL-treated mice and plugged orifice in *APOE*^–/–^ mice. The black dotted line shows dilated central ducts. Scale bars: 50 μm.

To image and evaluate MGs, eyelids were rapidly removed from age-matched WT mice, *ApoE^–/–^* mice, and IPL-treated mice. Images showed that *ApoE^–/–^* mice had great morphological hypertrophy in partial MGs with orifice obstruction. However, after IPL treatment, the plugged orifices reopened and MG hypertrophy was reduced (Figure 2A). To further confirm these changes, we performed H&E staining on the upper eyelids. Histological observations revealed that IPL treatment resulted in reopening of the plugged orifices (Figure 2B).

### Intense Pulsed Light Treatment Changes Lipid Accumulation and Mitochondrial Ultrastructure in Meibomian Glands

**FIGURE 3:**
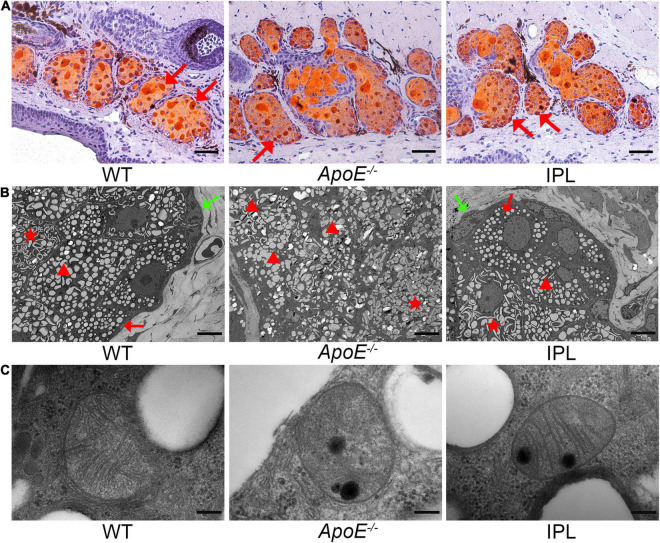
Lipid droplets and mitochondrial ultrastructural changes after IPL treatment. **(A)** ORO staining showing increased hyperchromatic lipid droplets in the MG acini in IPL-treated mice. The red arrow indicates the lipid droplets. **(B)** Transmission electron microscopic images of lipid droplets in acinar cells. The green arrow indicates the absence of lipid droplets in the cytoplasm of undifferentiated cells. The red arrow indicates small, round and regular lipid droplets within cells in the early stages of differentiation. The triangle indicates large and irregular lipid droplets in the middle or late stages of differentiation. The star indicates enlarged and fused lipid droplets within differentiated cells. **(C)** Transmission electron microscopic images of mitochondrial ultrastructure in MG acinar cells. Scale bars: 50 μm **(A)**; 5 μm **(B)**; 200 nm **(C)**.

ORO staining was performed to observe changes in lipid accumulation after IPL treatment. As shown in Figure 3A, fewer lipid droplets were seen in the acini of MGs from *ApoE^–/–^* mice than those from WT mice, while IPL treatment led to more hyperchromatic lipid droplets accumulating in the acini, although not as much as in WT mice. To better understand the changes of lipid droplets in acinar cells, we observed them under a transmission electron microscope and found that the cytoplasm of the acinar cells in *ApoE^–/–^* mice contained a large number of enlarged lipid droplets with irregular shape, with only a few small and regular lipid droplets (Figure 3B), suggesting that the *ApoE^–/–^* acinar peripheral cells were in the middle or late stages of differentiation (28). In contrast, there were no lipid droplets, or small and round lipid droplets distributed in acinar basal cells of WT mice and IPL-treated mice, indicating that the cells in the acinar basal layer of WT mice and IPL-treated mice were either undifferentiated or in the early stages of differentiation. Compared with the organized and dense cristae in mitochondria of WT mice, mitochondria of *ApoE^–/–^* mice were swollen, and the cristae were ruptured and disappeared, whereas mitochondrial injury was alleviated in the IPL-treated group, in which cristae were regularly arranged, but relatively sparse (Figure 3C).

### Intense Pulsed Light Treatment Decreases the Levels of Proinflammatory Cytokines and Downregulates the Nuclear Factor Kappa B Signaling Pathway

**FIGURE 4:**
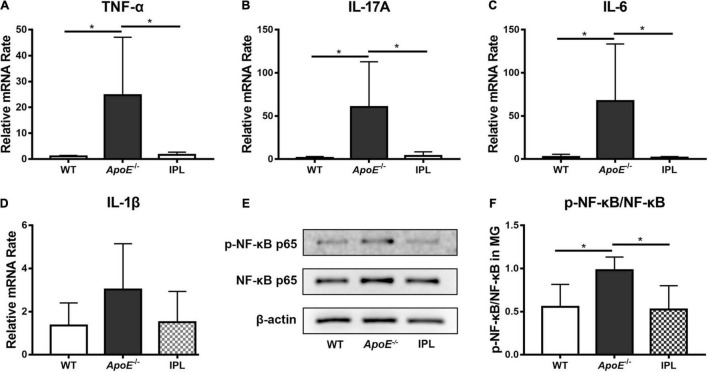
Effects of IPL treatment on inflammation in MGs. **(A–D)** The relative mRNA levels of TNF-α, IL-17A, IL-6, and IL-1β (*n* = 5–6/group). **(E)** Western blot result of p-NF-κB p65 and NF-κB p65. **(F)** Relative p-NF-κB p65 expression (*n* = 4/group) in MGs. *p < 0.05.

We examined the levels of TNF-α, IL-17A, IL-6, and IL-1β in MGs by qRT–PCR to observe whether IPL affected the expression of inflammatory factors. The results showed that IPL treatment significantly decreased the mRNA levels of TNF-α, IL-17A and IL-6 compared with *ApoE^–/–^* mice (*p* < 0.05), and the levels approached those observed in WT mice (Figures 4A–C). Although IL-1β showed no significant change, IPL-treated mice exhibited a trend toward reduced level (Figure 4D). Western blot showed that the expression of p-NF-κB p65 was downregulated in IPL-treated mice compared with *ApoE^–/–^* mice (*p* < 0.05) (Figures 4E,F). The results indicated that IPL treatment decreased the levels of inflammatory factors, in part by inactivating the NF-κB pathway.

### Intense Pulsed Light Treatment Increases the Proliferation of Acinar Basal Cells and Decreases Hyperkeratinization of the Ductal Epithelium

**FIGURE 5:**
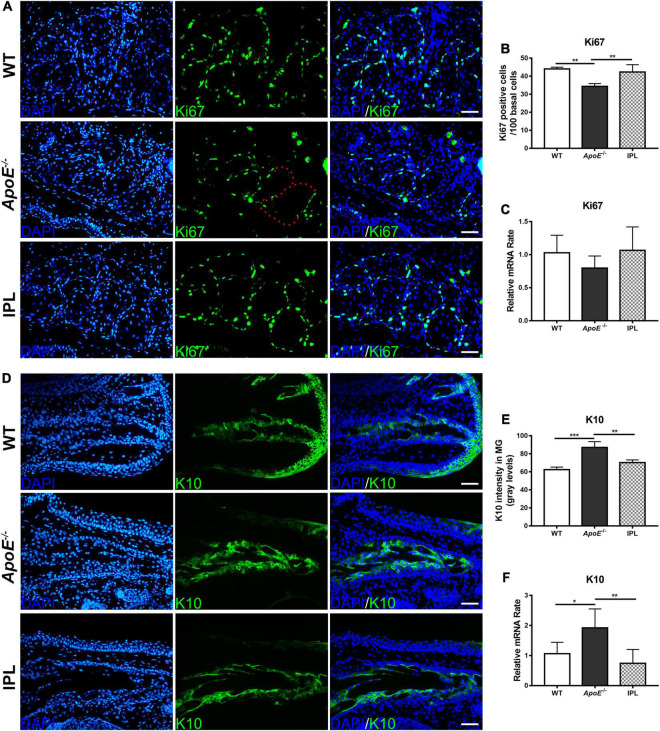
Increased proliferation and decreased keratinization in MGs after IPL treatment. **(A)** Images of Ki67 immunofluorescence staining. The red dotted line indicates that basal cells were Ki67 negative. **(B)** The counting number of Ki67-positive basal cells in MGs (*n* = 3/group). **(C)** Ki67 gene expression (*n* = 5–6/group). **(D)** Images of K10 immunofluorescence staining. **(E)** Quantification of K10 expression in the ducts of MGs (*n* = 3/group). **(F)** K10 gene expression (*n* = 5–6/group). *p < 0.05, **p < 0.01, ***p < 0.001. Scale bars: 50 μm.

The proliferation rate of basal cells was evaluated by immunofluorescence analysis of Ki67, a cell proliferation marker (29). The immunostaining results showed that Ki67-positive cells in the basal layer of acini of *ApoE^–/–^* mice were greatly reduced, while after IPL treatment Ki67-positive cells increased and were densely arranged at the edge of acinus (Figure 5A). To confirm this observation, we counted the number of Ki67-positive cells and found that three IPL treatments greatly improved the proliferation rate of acinar basal cells (*P* < 0.01) (Figure 5B). Although qRT–PCR showed no significant changes in the Ki67 mRNA levels in MGs, IPL-treated mice showed a trend toward increased Ki67 mRNA levels in comparison with *ApoE^–/–^* mice (Figure 5C).

K10 is regarded as a keratinization marker because it is expressed in terminally differentiated keratinocytes (30). Immunofluorescence staining showed that K10 was expressed in the ductal epithelia, and the fluorescence intensities of K10 in the *ApoE^–/–^* mice were elevated (*p* < 0.001). Similar to that in WT mice, the expression of K10 in the IPL-treated group was reduced (*p* < 0.01) (Figures 5D,E). qRT-PCR result further confirmed that the increased K10 gene expression in *ApoE^–/–^* mice was decreased after IPL treatment (*p* < 0.01) (Figure 5F).

### Intense Pulsed Light Treatment Influences Oxidative Stress and Apoptosis in Meibomian Glands

**FIGURE 6:**
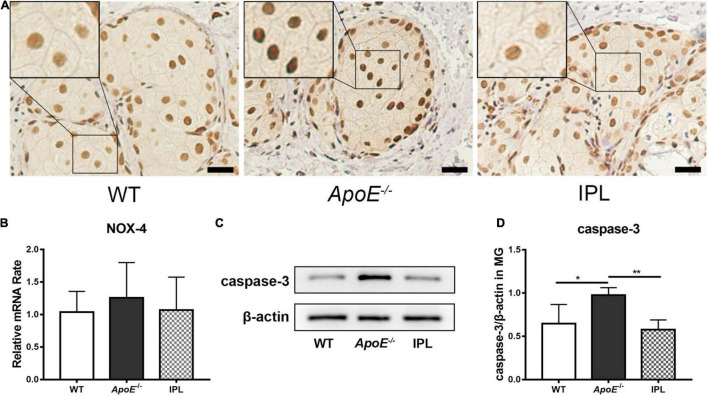
Downregulation of oxidative stress and apoptosis after IPL treatment. **(A)** immunohistochemical staining of NOX-4. The black frame indicates relatively decreased nuclear staining of the oxidative stress marker NOX-4 in IPL-treated mice compared with *APOE*^–/–^ mice. **(B)** NOX-4 gene expression (*n* = 5–6/group). **(C)** Western blot result of caspase-3. **(D)** Relative expression of caspase-3 (*n* = 4/group) in MGs. *p < 0.05, **p < 0.01; Scale bars: 50 μm.

NOX-4, a member of NOX family, is involved in the generation of reactive oxygen species (ROS) (31). The nuclear staining of NOX-4 was reduced in the acinar epithelial cells of IPL-treated mice when compared with those from *ApoE^–/–^* mice, but at approximately the same level as WT mice (Figure 6A). However, no difference in NOX-4 mRNA levels was observed between three groups (Figure 6B). Caspase-3, a biomarker of apoptosis, was detected by western blot, and the results showed that IPL reduced caspase-3 in MGs compared to those in *ApoE^–/–^* mice (*p* < 0.01) (Figures 6C,D), indicating that three courses of IPL treatment reduced cell death in the MGs of *ApoE^–/–^* mice.

## Discussion

**FIGURE 7:**
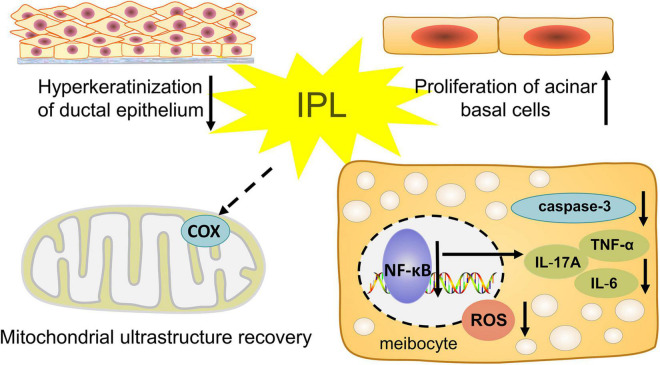
Illustration of the hypothetical mechanisms of indirect effects of IPL on MGD.

In this study, we observed the influence of IPL on morphological and pathological changes in the MGs. We chose *ApoE^–/–^* mice as our model, which developed hyperlipidemia that induced the occurrence and pathological course of MGD. Our results show that indirect irradiation via IPL to MGs reduces the severity of orifice plugging in *ApoE^–/–^* mice. In addition, our results demonstrate the anti-inflammatory effect of IPL, which may delay the progression of MGD. Indirect IPL irradiation also improves the biological function of MGs, including increased proliferation of MG basal cells and decreased oxidative stress (Figure 7). These results provide strong evidence that indirect irradiation via IPL is able to protect remote tissues- MGs and mitigate the development of MGD.

Although there have been many clinical studies on IPL treatment, and the main clinical assessments are the effect of IPL treatment on patient symptoms and tear film stability (32). The main criteria when evaluating the treatment efficacy of IPL on MGs is meibomian gland expression and its morphology, but the effect of IPL on MG itself cannot be truly observed. To date, the mechanisms of IPL treatment of MGD are elusive. In our study, we found that compared with *ApoE^–/–^* mice, IPL-treated mice had less MG obstruction, and improved lid margin and glandular morphology, which suggested the effect of IPL on MGs and the therapeutic effect on MGD. Furthermore, we explored the indirect effect of IPL, which is based on clinical practice for the use of external eye shields to cover and protect the eyes and eyelashes but at the same time covering MGs (9, 33). In our research, only the skin below the lower eyelids was irradiated by IPL, but beneficial effects were observed in both the upper and lower eyelids, consistent with the clinical therapeutic effect of IPL on patients with MGD even if there was no direct exposure of MGs to light (19, 20). In fact, the indirect effect of light therapy has been widely observed in PBM. Indirect effects of PBM have been observed in other fields, including promoting the wounds and burns healing, mitigating diabetes-induced retinopathy and exerting neuroprotective effects far from the irradiation site (21, 34–36). The phenomenon in which localized treatments induces beneficial effects in other distant tissues is termed the “abscopal effect.” Given that IPL is a PBM therapy, its treatment on MGD is low in energy, and the spectrum of IPL includes red and NIR light that function as PBM, the indirect effects of PBM may also explain the indirect effects of IPL.

Studies have shown that localized radiation therapy in patients with metastatic cancer sometimes leads to diminution of metastases far from the irradiation site, this abscopal effect thought to be mediated by systemic cytokines and/or immune responses (37). Interestingly, in our study, we found that indirect IPL irradiation significantly decreased proinflammatory cytokine expression, alleviated edema of the lid margin and reduced the fluorescein staining score. Therefore, we hypothesized that the indirect effect of IPL on MGD may also involve changes in inflammatory pathways. The anti-inflammatory effect of IPL has been indicated in clinical trials (10, 38, 39). Our previous study reported that IPL treatment reduced proinflammatory cytokines in the tears of MGD individuals (10). IPL is known to reduce telangiectasia to prevent the leakage of cytokines through the facial artery and orbital vessels, which are the major sources of inflammation in MGs (40). Furthermore, underlying the IPL-mediated downregulation of proinflammatory cytokines, we found that IPL treatment also notably decreased the activation of NF-κB, a transcription factor related to inducing inflammation. NF-κB can promote immune responses by controlling the expression of inflammation-related genes, including cytokines. When the NF-κB pathway is activated, the p65 subunit separates from the inhibitor of nuclear factor-κB (IκB) and transfers from the cytoplasm to the nucleus to regulate the expression of inflammation-related genes. Activation of target genes requires phosphorylation of the p65 subunit and its nuclear translocation (41, 42). In our study, we found that the expression of p-NF-κB p65 was downregulated in IPL-treated mice. Our results suggest that indirect IPL irradiation may affect the NF-κB signaling pathway to alter the inflammatory response. To further verify this result, we will explore the nuclear translocation of NF-κB *in vitro*. Previous studies found that the expressions of nucleotide-binding oligomerization domain-like receptor family pyrin domain containing three (NLRP3) inflammasome and its downstream inflammatory factors—caspase-1, IL-1β, and IL-18 were upregulated in dry eye patients (43) and in desiccating stress-induced murine dry eye models (44). These findings suggested that the inflammasome and its downstream inflammatory factors were involved in the development of ocular surface inflammation in dry eye. In addition, the activation of NF-κB is important for initiating NLRP3 priming (45). In future experiments, we will conduct in-depth research on whether IPL influences the expression of NLRP3 inflammasome.

In this study, clinical and histological observations showed that IPL treatment significantly reduced toothpaste-like meibum in MG orifices and ductal dilation, while K10 expression was downregulated in MGs. The melting point of MG secretions from MGD eyes was 3°C higher than that from normal eyes (46), and the energy delivered by IPL irradiation can liquefy the inspissated meibum (47, 48), consequently relieving ductal obstruction and releasing meibum into the tear film (8). In addition, ductal hyperkeratinization has been reported to be the main pathological change in obstructive MGD, resulting in increased viscosity of meibum and degenerative gland dilatation and atrophy (1, 49). Previous studies have reported that proinflammatory cytokines can induce ductal hyperkeratinization and obstruction of MG orifices, and blocking cytokines protected mice from the development of obstruction (50, 51). Thus, it is possible that plugged orifices and the hyperkeratinization of MG ducts are decreased because of the reduction in proinflammatory cytokines by the indirect effect of IPL. This finding also indicates that IPL treatment can prevent MG hyperkeratinization to disrupt a vicious cycle of pathological change.

In addition to anti-inflammatory effect of indirect IPL irradiation, we also found that IPL can influence the biological function of MG epithelial cells. We found the increased proliferation rate of acinar basal cells and the improvements in lipid droplets in IPL-treated mice. This result might explain why MG dropout showed improvements in IPL-treated patients (52–55). It has been reported that IPL can induce the proliferation of primary fibroblasts and regulate the hair growth cycle (56, 57). Systemic effects of PBM with red and NIR for topical treatment have been reported (21). Low-level laser therapy has systemic effects on wounds away from the irradiation site to promote their healing (35). In a rat myocardial infarction model, NIR light exposure to the bone marrow stimulates c-kit-positive cells and recruits them specifically to injury site to reduce myocardial infarct size (22). The indirect effect of IPL on MGD is similar to this progress, and the light spectrum of IPL includes that of red to NIR spectrum. Therefore, it is thought that indirect effect of IPL on MG epithelial cells occurs through the PBM mechanism, and the mechanism has yet to be elucidated.

Oxidative stress has been reported to be involved in the pathophysiological changes in MGD (24). The excess production of ROS induces oxidative damage to cellular macromolecules, leading to organelle dysregulation that eventually triggers apoptosis in cells (58). Here, we found that the indirect irradiation of MGD with IPL downregulated ROS-associated NOX-4 in *ApoE^–/–^* mice whose meibocytes were subjected to oxidative stress. In addition, the morphology and function of mitochondria improved and apoptosis decreased. In the UVB-induced photoaged phenotype of skin cells, IPL also reduced intracellular oxidative stress, which is consistent with our results (59). Although PBM increases ROS under normal conditions, PBM delivered to cells that are already in a state of oxidative stress induces the reduction of high ROS concentrations and suppression of apoptosis (60). In the future, we will detect ROS production to confirm the result and continue to explore the signaling pathway related to ROS. Given the characteristics of the light spectrum of IPL, we hypothesize that the photons from IPL may be absorbed by cytochrome C oxidase (COX), a key enzyme in the mitochondrial electron transport chain responsible for the beneficial effects of PBM. This process can increase the generation of ATP and change the levels of ROS (61, 62). More research is necessary to confirm our hypothesis and further explore the antioxidant mechanism of IPL.

In conclusion, the present study demonstrates that IPL irradiation can improve the structure and function of MGs and modify the disease progression of MGD in a mouse model, which may be related to the indirect effects of PBM.

## Data Availability Statement

The original contributions presented in the study are included in the article/Supplementary Material, further inquiries can be directed to the corresponding authors.

## Ethics Statement

The animal study was reviewed and approved by the Laboratory Animal Ethics Committee of Peking University First Hospital.

## Author Contributions

XY and SY conceived and designed the study, and reviewed and revised the manuscript. LX and WS performed the experiment and wrote the manuscript. WD assessed the clinical parameters. YL, SC, XS, MH, YC, and YG were involved in data analysis and interpretation. All authors contributed to the article and approved the submitted version.

## Conflict of Interest

The authors declare that the research was conducted in the absence of any commercial or financial relationships that could be construed as a potential conflict of interest.

## Publisher’s Note

All claims expressed in this article are solely those of the authors and do not necessarily represent those of their affiliated organizations, or those of the publisher, the editors and the reviewers. Any product that may be evaluated in this article, or claim that may be made by its manufacturer, is not guaranteed or endorsed by the publisher.
