# Supplementary material for: Indirect Application of Intense Pulsed Light Induces Therapeutic Effects on Experimental Murine Meibomian Gland Dysfunction
Source: Front Med (Lausanne). 2022 Jun 2;9:923280. doi: 10.3389/fmed.2022.923280 (PMC9201038; doi:10.3389/fmed.2022.923280)
Supplement: Supplementary file 1 [file Data_Sheet_1.docx]

Supplementary Material

**Supplementary Table 1** | Analysis of the number of plugged orifices of meibomian glands in the upper eyelids

|  | WT group | *ApoE^-/-^* mice | IPL-treated group | *P* |
| --- | --- | --- | --- | --- |
| T1 | 4.1±2.3 | 5.7±1.9 | 3.3±1.3^#^ | **0.025** |
| T2 | 4.1±1.9 | 5.7±1.3 | 3.8±2.1 | 0.054 |
| T3 | 4.4±1.3 | 6.7±1.5^*^ | 3.8±1.2^#^ | **<0.001** |

T1 means two weeks after the first IPL treatment, T2 means two weeks after the second IPL treatment, and T3 means two weeks after the third IPL treatment.

*P* represents the comparison results of three groups; In post hoc results, ^*^ represents *p* < 0.05 compared with WT mice; ^#^ represents *p* < 0.05 compared with *ApoE^-/-^* mice.

**Supplementary Table 2** | Analysis of the number of plugged orifices of meibomian glands in the lower eyelids

|  | WT group | *ApoE^-/-^* mice | IPL-treated group | *P* |
| --- | --- | --- | --- | --- |
| T1 | 4.7±1.4 | 5.2±1.8 | 3.1±1.2^#^ | **0.010** |
| T2 | 4.8±1.6 | 5.7±1.6 | 3.7±1.6^#^ | **0.030** |
| T3 | 5.1±1.4 | 6.6±1.1^*^ | 4.3±1.2^#^ | **0.001** |

T1 means two weeks after the first IPL treatment, T2 means two weeks after the second IPL treatment, and T3 means two weeks after the third IPL treatment.

*P* represents the comparison results of three groups; In post hoc results, ^*^ represents *p* < 0.05 compared with WT mice; ^#^ represents *p* < 0.05 compared with *ApoE^-/-^* mice.

**Supplementary Table 3** | Analysis of corneal fluorescein staining scores

|  | WT group | *ApoE^-/-^* mice | IPL-treated group | *P* |
| --- | --- | --- | --- | --- |
| T1 | 10.2±1.2 | 13.2±0.9^*^ | 9.3±1.7^#^ | **<0.001** |
| T2 | 10.2±1.6 | 13.0±0.8^*^ | 10.4±1.1^#^ | **<0.001** |
| T3 | 10.4±1.3 | 13.2±1.4^*^ | 10.7±1.8^#^ | **<0.001** |

T1 means two weeks after the first IPL treatment, T2 means two weeks after the second IPL treatment, and T3 means two weeks after the third IPL treatment.

*P* represents the comparison results of three groups; In post hoc results, ^*^ represents *p* < 0.05 compared with WT mice; ^#^ represents *p* < 0.05 compared with *ApoE^-/-^* mice.
